# Scallop mantle extract inhibits insulin signaling in HepG2 cells

**DOI:** 10.1002/fsn3.1061

**Published:** 2019-05-08

**Authors:** Takahide Kariya, Koto Takahashi, Daisuke Itagaki, Yasushi Hasegawa

**Affiliations:** ^1^ College of Environmental Technology Muroran Institute of Technology Muroran Japan

**Keywords:** HepG2 cell, insulin signaling, scallop mantle, toxin

## Abstract

Scallops are important marine products in Hokkaido, Japan. Not only scallop adductor muscle but also mantle is often eaten at sashimi or smoking in Japan. We showed previously that feeding the scallop mantle epithelial cell layer causes an increase in serum glucose concentration and the death of rats. To clarify the mechanism of glucose metabolism disorder by mantle epithelial cell layer, we investigated whether extracts from mantle tissue (mantle extract) induce insulin resistance using HepG2 cells. Mantle extract suppressed insulin‐stimulated phosphorylation of Akt, key protein which is involved in insulin signaling. In addition, treatment of HepG2 cells with mantle extract decreased significantly glycogen content and mRNA expression levels of glucose‐6‐phosphatase (G6Pase) and phosphoenolpyruvate carboxykinase (PEPCK) involved in gluconeogenesis, suggesting that mantle extract inhibits insulin signaling. These results show that mantle extract inhibits insulin signaling in HepG2 cells, suggesting that an increase in serum glucose concentration in vivo may be due to the inhibition of insulin signaling.

## 1 INTRODUCTION

Shellfish such as scallops, clams, and mussels accumulate marine toxins that are produced by algae such as toxic dinoflagellate. Toxification of shellfish gives large damage to seafood and fishing industries. Two types of toxicity have been detected in shellfish that was harvested in Japan: One is paralytic shellfish poisoning (PSP) and the other is diarrhetic shellfish poisoning (DSP; Farabegoli, Blanco, Rodríguez, Vieites, & Cabado, 2018; Smith & Swoboda, 2018). We reported previously that feeding the scallop mantle epithelial cell layer causes an increase in serum glucose concentration and death of rats (Hasegawa, Itagaki, Konno, & Hasegawa, 2018). In addition, we suggested that the toxic substance is unknown toxic one different from PSP and DSP toxins.

Insulin is a hormone, which is responsible for regulating blood glucose levels and keeping a steady blood glucose level. Insulin activates insulin receptors and causes tyrosine phosphorylation of insulin receptor substrate (IRS; Valverde et al., 2003). The phosphorylation activates phosphatidylinositol3 (PI3) kinase‐Akt pathway. Activated Akt (phosphorylated Akt) induces glycogen synthesis through phosphorylation (inactivation) of glycogen synthase kinase (GSK)‐3β (Beurel, Grieco, & Jope, 2015). Akt also suppresses gluconeogenesis by lowering the expression of its related enzymes in hepatocytes (Liu et al., 2015).

Insulin resistance is a status in which cells do not respond properly to the insulin and causes high blood glucose concentration, leading to type 2 diabetes. Insulin resistance is a deficit in signal transduction from insulin such as inactivation of PI3 kinase‐Akt signaling pathway. While the exact cause of insulin resistance is still not understood, factors such as obesity, inflammation, chronic stress, and lack of physical activity have been reported (Sanghez et al., 2016; Shoelson, Herrero, & Naaz, 2007). There have been many studies about the pathway causing insulin resistance. Endoplasmic reticulum (ER) stress has been reported to inhibit insulin signaling through pathways such as inactivation of IRS by activation of C‐Jun N‐terminal kinase (JNK; Castro et al., 2013; Kaneto, Nakatani, & Matsuhisa, 2004). Obesity and inflammation increase the expression of suppressor of cytokine signaling (SOCS) protein 1 and protein 3 in liver and inhibits tyrosine phosphorylation of IRS, leading to the insulin resistance (Rui, Yuan, Frantz, Shoelson, & White, 2002; Ueki, Kondo, & Kahn, 2004).

The human hepatoma cell line HepG2 has extensively been used to investigate hyperglycemia and diabetes because these cells exhibit many functions of normal human hepatocytes (Hu et al., 2014; Vidyashankar, Varma, & Patki, Varma & Patki, 2013; Zang et al., 2004). In this study, we studied whether mantle extract causes insulin resistance in HepG2 cells for clarifying the action mechanism increasing serum glucose concentration.

## 2 MATERIALS AND METHODS

### 2.1 Materials

Scallops (*Patinopecten yessoensis*), which were harvested from Mutsu Bay, Aomori, Japan, were purchased on the market. Mantle including epithelial cell layer was prepared from the scallops. β‐Actin, Akt, S473‐phosphorylated Akt (p‐Akt), JNK, T183‐phosphorylated JNK (p‐JNK), GSK‐3β, S9‐phosphorylated GSK‐3β (p‐GSK‐3β), IRS‐1, or S307‐phosphorylated IRS‐1(p‐IRS) was purchased from Biorbyt.

### 2.2 Extract from the scallop mantle tissue

Extract of mantle tissue including mantle epithelial cell layer was prepared as described previously (Hasegawa et al., 2018). Mantle tissue was lyophilized and homogenized in deionized water. After centrifugation at 12,000 × *g* for 15 min, the supernatant was used as the mantle extract.

### 2.3 Cell culture and viability assay

Human hepatoma HepG2 cells were purchased from RIKEN Cell Bank. HepG2 cells were maintained in modified Eagle's medium (MEM) and 10% fetal calf serum. Cells were seeded into a 96‐well plate at a density of 4 × 10^3^ cells/well and cultured overnight. After cells were treated with various concentrations of mantle extract for 48 hr, cell viability was estimated by the 3‐(4,5‐dimethylthiazol‐2‐yl)‐2,5‐diphenyltetrazolium bromide (MTT) assay (Manthorpe, Fagnani, Skaper, & Varon, 1986).

### 2.4 Glycogen content

Glycogen content in HepG2 cells was examined in the presence or absence of mantle extract according to a sight modification of the method described previously (Hasegawa et al., 2018). Briefly, HepG2 cells were seeded on a 6‐well plate at a density of 1 × 10^5^ cells/well. After 24 hr, culture medium was exchanged to serum‐free medium and mantle extract was added to the culture medium at the indicated concentration. After cells were treated with 100 nM insulin for 30 min, cells were collected and trichloroacetic acid was added to be 10%. After incubating the solution at 100°C for 20 min, centrifugation at 14,000 *g* for 5 min at 4°C was performed and the supernatant was collected. Ethanol was added to be 80% and centrifuged at 14,000 *g* for 15 min at 4°C. The precipitation was dried and dissolved at deionized water, and anthrone reagent was added. After treatment at 100°C for 10 min, absorbance at 620 nm was measured.

### 2.5 Semi‐quantitative reverse transcription polymerase chain reaction analysis

After HepG2 cells were treated with insulin in the absence or presence of the mantle extract for 30 min, total RNA was prepared using an RNAiso Plus (Takara), as per the manufacturer's protocol. Total RNA was also prepared from liver tissues of mice fed control diet or mantle diet as well. To perform semi‐quantitative reverse transcription polymerase chain reaction (RT‐PCR), first‐strand complementary DNA (cDNA) was synthesized using oligo (dT) primer and PCR was carried out using specific primers. The specific forward and reverse primers are shown in Table 1. The intensities of the bands of the PCR products were quantitated using ImageJ software and normalized with respect to β‐actin. The PCR cycles were selected on the basis of the relationship between the number of cycles and amount of PCR product.

**Table 1:** Primer sequences used in semi‐quantitative RT‐PCR

Gene	Forward primer	Reverse primer
β‐Actin	5′‐CATCCGCAAAGACCTGTACG‐3′	5′‐CCTGCTTGCTGATCCACATC‐3′
G6Pase	5′‐ATTGACACCACACCCTTTGC‐3′	5′‐GACGTAGAAGACCAGCTCGA‐3′
SOCS	5′‐CACCTACTGAACCCTCCTCC‐3′	5′‐AGAGATGCT GAAGAGTGGCC‐3′
PEPCK	5′‐CTGGGAAGGCATTGATGAGC‐3′	5′‐CGGCCTCCAAAGATAATGCC‐3′
CHOP	5′‐AGGGAGAACCAGGAAACGGAAACA‐3′	5′‐TCCTGCTTGAGCCGTTCATTCTCT‐3′
Spiced Xbp‐1	5′‐TGTCACCCCTCCAGAACATC‐3′	5′‐AAGGGAGGCTGGTAAGGAAC‐3′
GRP78	5′‐CGGTCTACTATGAAGCCCGT‐3′	5′‐CATCTGGGTTTATGCCACGG‐3′

### 2.6 Western blotting

After HepG2 cells were treated with insulin in the absence or presence of the indicated concentrations of mantle extract for 30 min, cells were homogenized in a solution containing 0.2% SDS, 20 mM Tris‐HCl (pH 7.5), and bromophenol blue. After SDS polyacrylamide gel electrophoresis (Laemmli, 1970), the proteins were electrotransferred onto a polyvinylidene difluoride (PVDF) membrane. After blocking the membrane with 5% skim milk (w/v) in a solution containing 0.5 M NaCl, 20 mM Tris‐HCl (pH 7.5), and 0.05% Tween 20 (solution A) for 2–6 hr at room temperature, antibodies against β‐actin, Akt, p‐Akt, JNK, p‐JNK, GSK‐3β, p‐GSK‐3β, IRS‐1, or p‐IRS‐1 were incubated overnight. After washing with solution A, the membrane was treated with an alkaline phosphatase‐conjugated secondary antibody for 2 hr and developed with 5‐bromo‐4‐chloro‐3‐indolyl phosphate and nitroblue tetrazolium. The band intensities were estimated using Image J.

### 2.7 Statistical analysis

Each experiment was performed at least three times. Data were expressed as the mean and the standard deviation (*SD*). The data were analyzed using Student's *t* test.

## 3 RESULTS

### 3.1 Effects of mantle extract on insulin signaling in HepG2 cells

We showed previously that intake of mantle epithelial cell layer tissue finally increases serum glucose concentration (Hasegawa et al., 2018). In this study, we investigated the effect of mantle extract on insulin signaling using HepG2 cells for clarifying the cause of increased serum glucose concentration.

**Figure 1:**
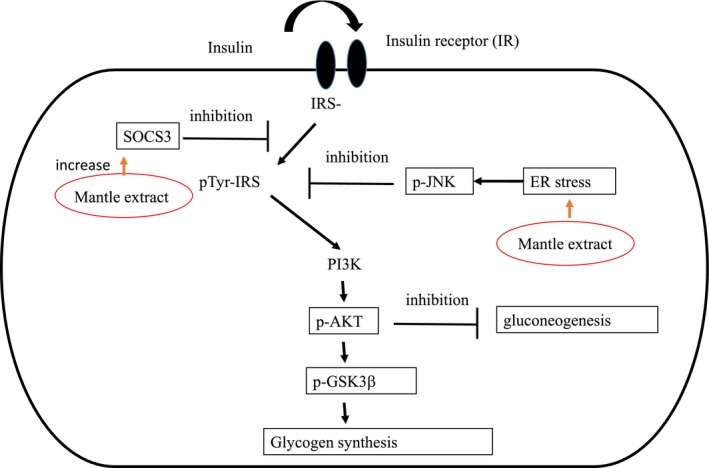
Signaling pathways after insulin stimulation. Inhibition of signaling was shown by lines ending in bars. Arrows show activation of pathways

**Figure 2:**
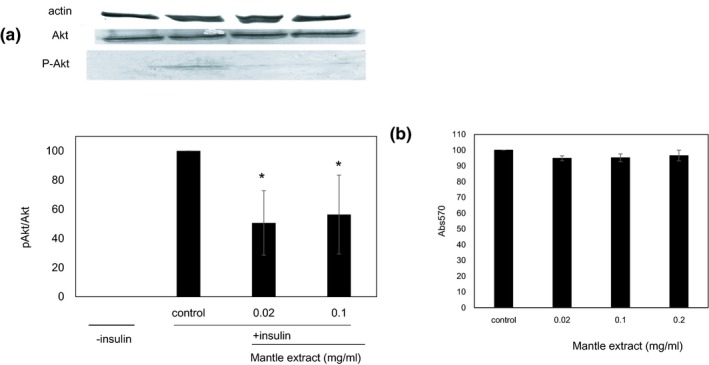
Effect of mantle extract on Akt phosphorylation after insulin stimulation. (a) HepG2 cells were treated with insulin in the absence or presence of the indicated concentrations of mantle extract for 30 min. Amount of nonphosphorylated and phosphorylated Akt was measured using Western blotting. Bars show *SD*. *p < 0.05 relative to control. (b) Toxicity of the mantle extract at the indicated concentrations was measured by MTT assay

Insulin stimulation of HepG2 cells induces tyrosine phosphorylation of Akt through binding to insulin receptor (Figure 1). We investigated whether treatment with mantle extract suppresses the phosphorylation of Akt in HepG2 cells. The phosphorylation of Akt was significantly inhibited in the presence of mantle extract at concentrations of 0.02 and 0.1 mg/ml (Figure 2). On the other hand, mantle extract did not show any toxicity at a concentration of 0.1 mg/ml. These results show that mantle extract inhibits insulin signaling.

**Figure 3:**
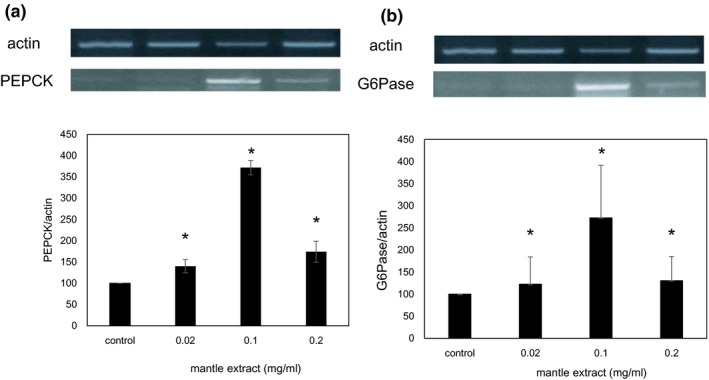
Effect of mantle extract on the mRNA expressions of G6Pase (a) and PEPCK (b). After HepG2 cells were treated with insulin in the absence or presence of the indicated concentrations of mantle extract for 30 min, semi‐quantitative RT‐PCR was performed. Bars show *SD*. *p < 0.05 relative to control

**Figure 4:**
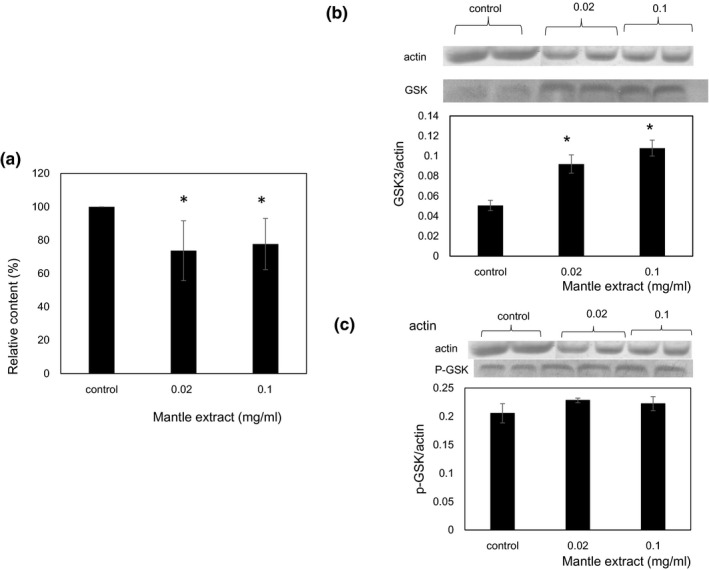
Effect of mantle extract on glycogen synthesis. (a) After HepG2 cells were treated with insulin in the absence or presence of the indicated concentrations of mantle extract for 30 min, cells were recovered and glycogen content was estimated. (b, c) Nonphosphorylated and phosphorylated GSK‐3β levels were measured using Western blotting. Bars show *SD*. *p < 0.05 relative to control

Insulin stimulation is known to suppress gluconeogenesis and promote glycogen synthesis in hepatocyte through phosphorylation of Akt (Figure 1). Therefore, inhibition of insulin signaling will promote gluconeogenesis and decrease glycogen content. In order to confirm that mantle extract inhibits insulin signaling pathway, we investigated the mRNA expression levels of two key enzymes of gluconeogenesis, G6Pase and PEPCK, by semi‐quantitative RT‐PCR. Mantle extract increased significantly G6Pase and PEPCK mRNA expression levels to about 2.5–3.5‐fold compared to the control (Figure 3), suggesting that mantle extract inhibits insulin signaling. Next, we investigated glycogen content in HepG2 cells. Treatment with mantle extract significantly decreased glycogen content to about 70% compared to that of the control (Figure 4). To confirm further this result, we measured the expression levels of phosphorylated GSK‐3β which regulates glycogen synthesis using Western blot. Mantle extract increased significantly the expression of unphosphorylated GSK‐3β level (Figure 4). Increase in unphosphorylated GSK‐3β decreases glycogen content through inactivation of glycogen synthase. This result also supports that mantle extract inhibits insulin signaling in HepG2 cells.

### 3.2 Mantle extract induces ER stress

**Figure 5:**
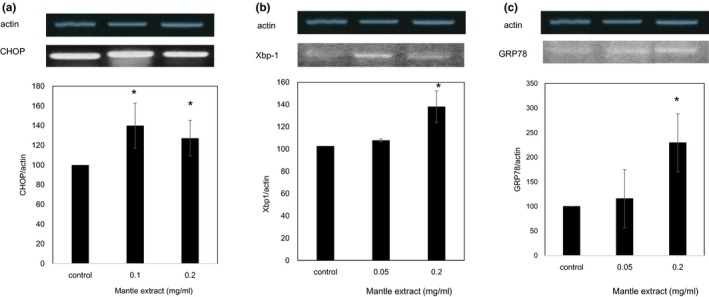
ER stress (a–c) by mantle extract. After HepG2 cells were treated with insulin in the absence or presence of the indicated concentrations of mantle extract for 30 min, semi‐quantitative RT‐PCR was performed using specific primers of CHOP (a), spliced Xbp‐1 (b), and GRP78 (c). Bars show *SD*. *p < 0.05 relative to control

**Figure 6:**
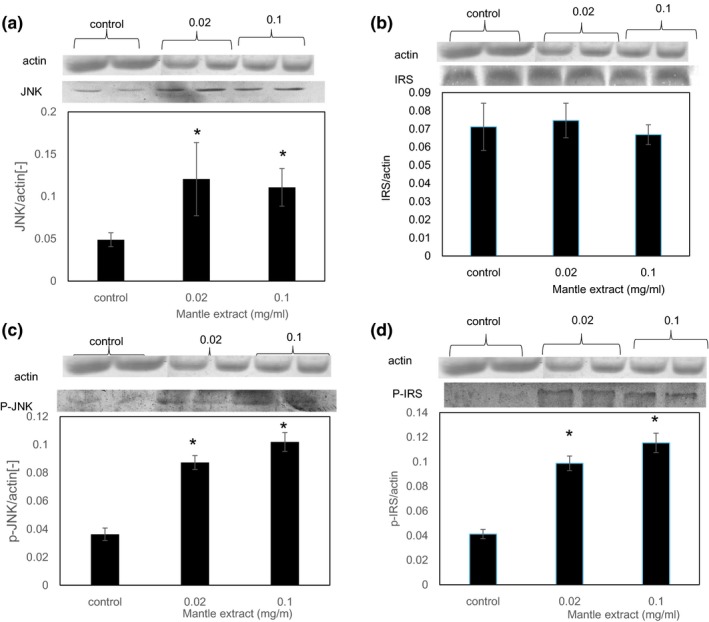
Expression levels of nonphosphorylated and phosphorylated JNK (a, b) and IRS‐1 (c, d). After HepG2 cells were treated with insulin in the absence or presence of the indicated concentrations of mantle extract for 30 min, Western blotting was performed. Bars show *SD*. *p < 0.05 relative to control

To clarify further the inhibitory mechanism of the insulin signaling by the mantle extract, we investigated mRNA expression levels of ER stress‐induced genes (Samali, FitzGerald, Deegan, & Gupta, 2010), which have been reported to causes insulin resistance (Figure 1). Addition of mantle extract increased mRNA expressions of ER stress markers, CHOP, GRP78, and spliced Xbp‐1 (Figure 5). In addition, the mantle extract increased the level of phosphorylated JNK, which is phosphorylated by ER stress, and increased serine phosphorylation of IRS (Figure 6). Phosphorylated JNK is known to promote serine phosphorylation of IRS which suppresses insulin signaling (Figure 1). These results suggest that mantle extract induces ER stress and leads to activation of JNK, resulting in inhibition of the insulin signaling.

**Figure 7:**
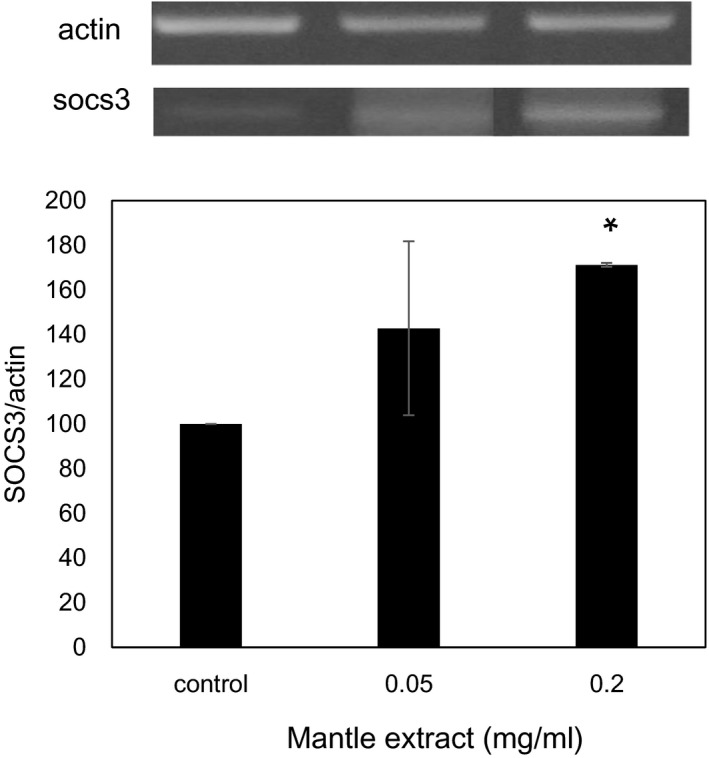
Effect of mantle extract on a mRNA expression of SOCS‐3. After HepG2 cells were treated with insulin in the absence or presence of the indicated concentrations of mantle extract for 30 min, semi‐quantitative RT‐PCR was performed using specific primers of SOCS‐3

Finally, we investigated the expression level of SOCS‐3 which suppresses tyrosine phosphorylation of IRS (Figure 1). Mantle extract increased significantly the expression of SOCS‐3 (Figure 7). These results suggest that mantle extract inhibits insulin signaling through ER stress and the increase in SOCS‐3 expression in HepG2 cells.

## 4 DISCUSSION

We previously found that feeding diets containing mantle epithelial cell layer caused an increase in serum glucose concentration and death of rats (Hasegawa et al., 2018). In this study, we studied whether mantle extract causes insulin resistance in HepG2 cells for clarifying the action mechanism increasing serum glucose concentration. We showed that mantle extract inhibited insulin signaling pathway by reducing phosphorylation of Akt and led to a decrease in glycogen content through GSK‐3β. In addition, the inhibition of Akt phosphorylation promoted gluconeogenesis by increasing the expression of PEPCK and G6Pase in HepG2 cells. Insulin resistance has been reported to be induced by several substances such as palmitate and glucosamine. Treatment of HepG2 cells with palmitate (0.5 mM; 0.12 mg/ml) induced inhibition of Akt phosphorylation, inactivation of GSK‐3β, and increased expression of PEPCK (Cang et al., 2016; Ishii, Maeda, Tani, & Akagawa, 2015; Tang et al., 2015; Yadollah, Kazemipour, Bakhtiyari, & Nazifi, 2017). Exposure of hepatocyte to high concentration of glucosamine (10 mM, 1.79 mg/ml) also suppressed the insulin response for Akt phosphorylation and stimulated the expression of G6Pase and PEPCK (Liu et al., 2015). The water‐soluble mantle extract inhibits insulin signaling at a concentration of 0.1–0.2 mg/ml. Mantle extract does not seem to contain high concentration of palmitate or glucosamine, suggesting that mantle extract contains the insulin signaling‐inhibiting substance different from palmitate and glucosamine.

Many studies have shown the link between ER stress and insulin resistance. ER stress causes insulin resistance through several pathways. ER stress activates JNK, leading to serine phosphorylation of IRS (Ozcan et al., 2004), which inhibits Akt phosphorylation and insulin signaling downstream in vivo. Lee et al. (2010) showed that transcription factor (CREBH) which is activated by ER stress induces transcription of G6Pase and PEPCK. Treatment with the mantle extract increased the expression of GRP78, CHOP, and spliced Xbp‐1, which are ER stress markers. In addition, the mantle extract increased phosphorylation of JNK and expression levels of G6Pase and PEPCK, suggesting that the mantle extract inhibits insulin signaling through ER stress and CREBH activation.

Insulin resistance is also caused by inflammatory mediators such as interleukin‐6 (IL‐6) (Senn et al., 2003; Ueki et al., 2004). When HepG2 cells were treated with IL‐6, the expression of SOCS‐3 mRNA is induced. Upregulation of SOCS‐3 in the liver of diabetic mice causes insulin resistance by inhibiting tyrosine phosphorylation of IRS. We found that treatment with the mantle extract increased the expression of SOCS‐3, suggesting that treatment with mantle extract may induce inflammatory cytokines in liver. Mantle extract may also inhibit insulin signaling through inflammation in addition to ER stress.

We showed that treatment of HepG2 cells with the mantle extract decreased their glycogen content and increased the mRNA levels of G6Pase and PEPCK. We had previously reported that feeding rats with the mantle tissue decreased the glycogen content in their livers (Hasegawa et al., 2018). We also observed that their livers had increased mRNA levels of G6Pase and PEPCK (T, Kariya, K, Takahashi, Y, Hasegawa, unpublished data). Taken together, these observations suggest that feeding rats with the mantle tissue may also inhibit insulin signaling pathway in their livers. However, it is unclear whether such toxicity also occurs in humans upon ingestion of the mantle tissue. Before investigating this possibility, we first need to isolate the toxic substance and clarify the dose–toxicity relationship and the underlying mechanism in rats. Currently, we are in the process of identifying this toxic substance and its action mechanism.

## 5 CONCLUSION

In this study, we studied whether mantle extract causes insulin resistance in HepG2 cells for clarifying the action mechanism increasing serum glucose concentration in vivo and showed that the mantle extract inhibits insulin signaling in HepG2 cells, suggesting that an increase in serum glucose concentration in vivo may be due to the inhibition of insulin signaling. Now, it remains unclear whether an insulin signaling‐inhibiting substance is a causal substance causing death of rat. Now, we are trying to identify the causal substance causing dead of rat and then clarify whether the substance inhibits the insulin signaling.

## CONFLICT OF INTEREST

The authors declare no conflict of interest.

## ETHICAL STATEMENT

This study does not involve any human nor animal testing.

## References

[fsn31061-bib-0001] Beurel, E. , Grieco, S. F. , & Jope, R. S. (2015). Glycogen synthase kinase‐3 (GSK3): Regulation, actions, and diseases. Pharmacology and Therapeutics, 148, 114–131. 10.1016/j.pharmthera.2014.11.016 25435019 PMC4340754

[fsn31061-bib-0002] Cang, X. , Wang, X. , Liu, P. , Wu, X. , Yan, J. , Chen, J. , …, Wang, X. (2016). PINK1 alleviates palmitate induced insulin resistance in HepG2 cells by suppressing ROS mediated MAPK pathways. Biochemical and Biophysical Research Communications, 478, 431–438. 10.1016/j.bbrc.2016.07.004 27423393

[fsn31061-bib-0003] Castro, G. , Areias, M. F. C. , Weissmann, L. , Quaresma, P. G. F. , Katashima, C. K. , Saad, M. J. A. , & Prada, P. O. (2013). Diet‐induced obesity induces endoplasmic reticulum stress and insulin resistance in the amygdala of rats. FEBS Open Bio, 3, 443–449. 10.1016/j.fob.2013.09.002 PMC3829990 24251109

[fsn31061-bib-0004] Farabegoli, F. , Blanco, L. , Rodríguez, L. P. , Vieites, J. M. , & Cabado, A. G. (2018). Phycotoxins in marine shellfish: Origin, occurrence and effects on humans. Marine Drugs, 16, 188 10.3390/md16060188 PMC6025170 29844286

[fsn31061-bib-0005] Hasegawa, Y. , Itagaki, D. , Konno, K. , & Hasegawa, C. (2018). Feeding of scallop mantle epithelial cell layer causes subacute toxicity against rodents. Fisheries Science, 84, 91–100. 10.1007/s12562-017-1156-3

[fsn31061-bib-0006] Hu, X. , Wang, S. , Xu, J. , Wang, D. B. , Chen, Y. , & Yang, G. Z. (2014). Triterpenoid saponins from *Stauntonia chinensis* ameliorate insulin resistance via the AMP‐activated protein kinase and IR/IRS‐1/PI3K/Akt pathways in insulin‐resistant HepG2 cells. International Journal of Molecular Sciences, 15, 10446–10458. 10.3390/ijms150610446 24918297 PMC4100161

[fsn31061-bib-0007] Ishii, M. , Maeda, A. , Tani, S. , & Akagawa, M. (2015). Palmitate induces insulin resistance in human HepG2 hepatocytes by enhancing ubiquitination and proteasomal degradation of key insulin signaling molecules. Archives of Biochemistry and Biophysics, 566, 26–35. 10.1016/j.abb.2014.12.009 25527164

[fsn31061-bib-0008] Kaneto, H. , Nakatani, Y. , & Matsuhisa, M. (2004). ER stress and the JNK pathway in insulin resistance. Gene Therapy and Molecular Biology, 8, 515–522.

[fsn31061-bib-0009] Laemmli, U. K. (1970). Cleavage of structural proteins during the assembly of the head of bacteriophage T4. Nature, 227, 680–685. 10.1038/227680a0 5432063

[fsn31061-bib-0010] Lee, M. W. , Chanda, D. , Yang, J. , Oh, H. , Kim, S. S. , Yoon, Y. S. , …, Koo, S. H. (2010). Regulation of hepatic gluconeogenesis by an ER‐bound transcription factor, CREBH. Cell Metabolism, 11, 331–339. 10.1016/j.cmet.2010.02.016 20374965

[fsn31061-bib-0011] Li, X. , Wang, Y. , Wang, H. , Huang, C. , Huang, Y. , & Li, J. (2015). Endoplasmic reticulum stress is the crossroads of autophagy, inflammation, and apoptosis signaling pathways and participates in liver fibrosis. Inflammation Research, 64, 2159–7. 10.1007/s00011-014-0772-y 25286903

[fsn31061-bib-0012] Liu, T. Y. , Shi, C. X. , Gao, R. , Sun, H. J. , Xiong, X. Q. , Ding, L. , …, Zhu, G. Q. (2015). Irisin inhibits hepatic gluconeogenesis and increases glycogen synthesis via the PI3K/Akt pathway in type 2 diabetic mice and hepatocytes. Clinical Science, 129, 839–850. 10.1042/CS20150009 26201094

[fsn31061-bib-0013] Manthorpe, M. , Fagnani, R. , Skaper, S. D. , & Varon, S. (1986). An automated colorimetric microassay for neuronotrophic factors. Brain Research, 390, 191–198. 10.1016/0165-3806(86)90208-7 3955369

[fsn31061-bib-0014] Ozcan, U. , Cao, Q. , Yilmaz, E. , Lee, A. H. , Iwakoshi, N. N. , Ozdelen, E. , …, Hotamisligil, G. S. (2004). Endoplasmic reticulum stress links obesity, insulin action, and type 2 diabetes. Science, 306, 457–461. 10.1126/science.1103160 15486293

[fsn31061-bib-0015] Rui, L. , Yuan, M. , Frantz, D. , Shoelson, S. , & White, M. F. (2002). SOCS‐1 and SOCS‐3 block insulin signaling by ubiquitin‐mediated degradation of IRS1 and IRS2. Journal of Biological Chemistry, 277, 42394–42398. 10.1074/jbc.C200444200 12228220

[fsn31061-bib-0016] Samali, A. , FitzGerald, U. , Deegan, S. , & Gupta, S. (2010). Methods for monitoring endoplasmic reticulum stress and the unfolded protein response. International Journal of Cell Biology, 2010, 2159–11, 10.1155/2010/830307 PMC2821749 20169136

[fsn31061-bib-0017] Sanghez, V. , Cubuk, C. , Sebastián‐Leon, P. , Carobbio, S. , Dopazo, J. , Vidal‐Puig, A. , & Bartolomucci, A. (2016). Chronic subordination stress selectively downregulates the insulin signaling pathway in liver and skeletal muscle but not in adipose tissue of male mice. Stress, 19, 214–224. 10.3109/10253890.2016.1151491 26946982 PMC4841025

[fsn31061-bib-0018] Senn, J. J. , Klover, P. J. , Nowak, I. A. , Zimmers, T. A. , Koniaris, L. G. , Furlanetto, R. W. , & Mooney, R. A. (2003). Suppressor of cytokine signaling‐3 (SOCS‐3), a potential mediator of interleukin‐6‐dependent insulin resistance in hepatocytes. Journal of Biological Chemistry, 278, 13740–13746. 10.1074/jbc.M210689200 12560330

[fsn31061-bib-0019] Shoelson, S. E. , Herrero, L. , & Naaz, A. (2007). Obesity, inflammation, and insulin resistance. Gastroenterology, 132, 2169–2180. 10.1053/j.gastro.2007.03.059 17498510

[fsn31061-bib-0020] Smith, M. E. , & Swoboda, H. D. (2018). Toxicity, shellfish. Treasure Island, FL: StatPearls Publishing. 29262048

[fsn31061-bib-0021] Tang, Z. , Zhang, W. , Wan, C. , Xu, G. , Nie, X. , Zhu, X. , …, Wang, C. (2015). TRAM1 protect HepG2 cells from palmitate induced insulin resistance through ER stress‐JNK pathway. Biochemical and Biophysical Research Communications, 457, 578–584. 10.1016/j.bbrc.2015.01.027 25600807

[fsn31061-bib-0022] Ueki, K. , Kondo, T. , & Kahn, C. R. (2004). Suppressor of cytokine signaling 1 (SOCS‐1) and SOCS‐3 cause insulin resistance through inhibition of tyrosine phosphorylation of insulin receptor substrate proteins by discrete mechanisms molecular and cellular biology. American Society for Microbiology, 24, 5434–5446. 10.1128/MCB.24.12.5434-5446.2004 PMC419873 15169905

[fsn31061-bib-0023] Valverde, A. M. , Burks, D. J. , Fabregat, I. , Fisher, T. L. , Carretero, J. , White, M. F. , & Benito, M. (2003). Molecular mechanisms of insulin resistance in IRS‐2‐deficient hepatocytes. Diabetes, 52, 2239–2248. 10.2337/diabetes.52.9.2239 12941762

[fsn31061-bib-0024] Vidyashankar, S. , Varma, R. S. , & Patki, P. S. (2013). Quercetin ameliorate insulin resistance and up‐regulates cellular antioxidants during oleic acid induced hepatic steatosis in HepG2 cells. Toxicology in Vitro, 27, 945–953. 10.1016/j.tiv.2013.01.014 23348005

[fsn31061-bib-0025] Yadollah, S. , Kazemipour, N. , Bakhtiyari, S. , & Nazifi, S. (2017). Palmitate‐induced insulin resistance is attenuated by Pioglitazone and EGCG through reducing the gluconeogenic key enzymes expression in HepG2 cells. Journal of Medicine and Life, 10, 244–249. 29362600 PMC5771254

[fsn31061-bib-0026] Zang, M. , Zuccollo, A. , Hou, X. , Nagata, D. , Walsh, K. , Herscovitz, H. , …, Cohe, R. A. (2004). AMP‐activated protein kinase is required for the lipid‐lowering effect of metformin in insulin‐resistant human HepG2 cells. Journal of Biological Chemistry, 279, 47898–47905. 10.1074/jbc.M408149200 15371448

